# Muscone/RI7217 co-modified upward messenger DTX liposomes enhanced permeability of blood-brain barrier and targeting glioma

**DOI:** 10.7150/thno.41322

**Published:** 2020-03-04

**Authors:** Shuangming Kang, Wenjuan Duan, Shangqian Zhang, Dawei Chen, Jianfang Feng, Na Qi

**Affiliations:** 1 Department of Pharmaceutics, Shenyang Pharmaceutical University, Shenyang, 110016, China; 2 Department of Pharmacy, Guilin Medical University, Guilin, 541004, China; 3 Department of Pharmacy ,Guangxi University of Chinese Medicine, Nanning, 530299, China

**Keywords:** glioma, blood-brain barrier, muscone, RI7217, liposome

## Abstract

**Rationale**: The dual-targeted drug delivery system was designed for enhancing permeation of the blood-brain barrier (BBB) and providing an anti-glioma effect. As transferrin receptor (TfR) is over-expressed by the brain capillary endothelial (hCMEC/D3) and glioma cells, a mouse monoclonal antibody, RI7217, with high affinity and selectivity for TfR, was used to study the brain targeted drug delivery system. Muscone, an ingredient of traditional Chinese medicine (TCM) musk, was used as the "guide" drug to probe the permeability of the BBB for drug delivery into the cerebrospinal fluid. This study investigated the combined effects of TCM aromatic resuscitation and modern receptor-targeted technology by the use of muscone/RI7217 co-modified docetaxel (DTX) liposomes for enhanced drug delivery to the brain for anti-glioma effect.

**Methods**: Cellular drug uptake from the formulations was determined using fluorescence microscopy and flow cytometry. The drug penetrating ability into tumor spheroids were visualized using confocal laser scanning microscopy (CLSM). *In vivo* glioma-targeting ability of formulations was evaluated using whole-body fluorescent imaging system. The survival curve study was performed to evaluate the anti-glioma effect of the formulations.

**Results**: The results showed that muscone and RI7217 co-modified DTX liposomes enhanced uptake into both hCMEC/D3 and U87-MG cells, increased penetration to the deep region of U87-MG tumor spheroids, improved brain targeting *in vivo* and prolonged survival time of nude mice bearing tumor.

**Conclusion**: Muscone and RI7217 co-modified DTX liposomes were found to show improved brain targeting and enhanced the efficacy of anti-glioma drug treatment *in vivo.*

## Introduction

Amongst brain tumors, gliomas are among the most common primary brain tumors. Owing to infiltrating growth, glioma is not easy to be completely removed by surgery 1. Postoperative chemotherapy played an important role at improving the therapeutic efficacy of treatment against glioma. Unfortunately, most potential chemotherapy drugs cannot cross the BBB 2. In fact, more than 98 % of small molecule drugs and all large molecule drugs are not taken up by the brain capillary endothelial cells so most of the potential therapeutic drugs cannot enter the brain for the treatment of glioma 3. Brain targeted drug delivery systems are designed to increase drug concentration in brain tissues and improve its therapeutic effect. Brain targeting drug delivery systems based on nanotechnology using nanoparticles have seen rapid development with good therapeutic successes 4, 5.

The TfR is over-expressed in brain capillary endothelial and glioma cells 6 and its natural ligand is transferrin (Tf). Some researchers have demonstrated that Tf can recognize TfR through receptor-mediated transcytosis and could help to increase brain uptake 7, 8. However, high levels of endogenous Tf could saturate TfR and limits the use of applications targeting ligands to Tf 9. Recent studies reported that rat anti-TfR monoclonal antibody binds preferentially to the epitope of TfR which differs from the Tf binding site 10 and therefore, does not compete with the natural ligands. The most mature anti-TfR monoclonal antibody used is the monoclonal antibody OX26 against rat TfR 11. However, OX26 only recognizes the rat TfR 12. RI7217, a mouse monoclonal antibody with high affinity for TfR, had been used in brain targeting drug delivery systems 13-15. Although with 86 % homology between mouse and human TfR carrier proteins at the amino acid level, it is noteworthy that RI7217 binds to human brain vascular endothelial cells more efficiently than mouse endothelial cells 10, 16.

Muscone, a musk ingredient, can facilitate the uptake of some drugs to the brain 17. Muscone was proposed to alter the permeability of BBB to boost its uptake and that of other drugs into the brain 18, 19. It was reported by Chen et al. 20 and Wang et al. 21 that muscone promoted drug passage through the BBB by inhibiting the expression of P-glycoprotein (P-gp) and matrix metalloproteinase-9 (MMP-9), thereby relaxing the tight junctions between epithelial cells.

Although muscone has been shown to promote drugs crossing the BBB 18-21, the transport of covalently conjugated muscone to nanocarriers across the BBB has not been reported.

**Scheme 1:**
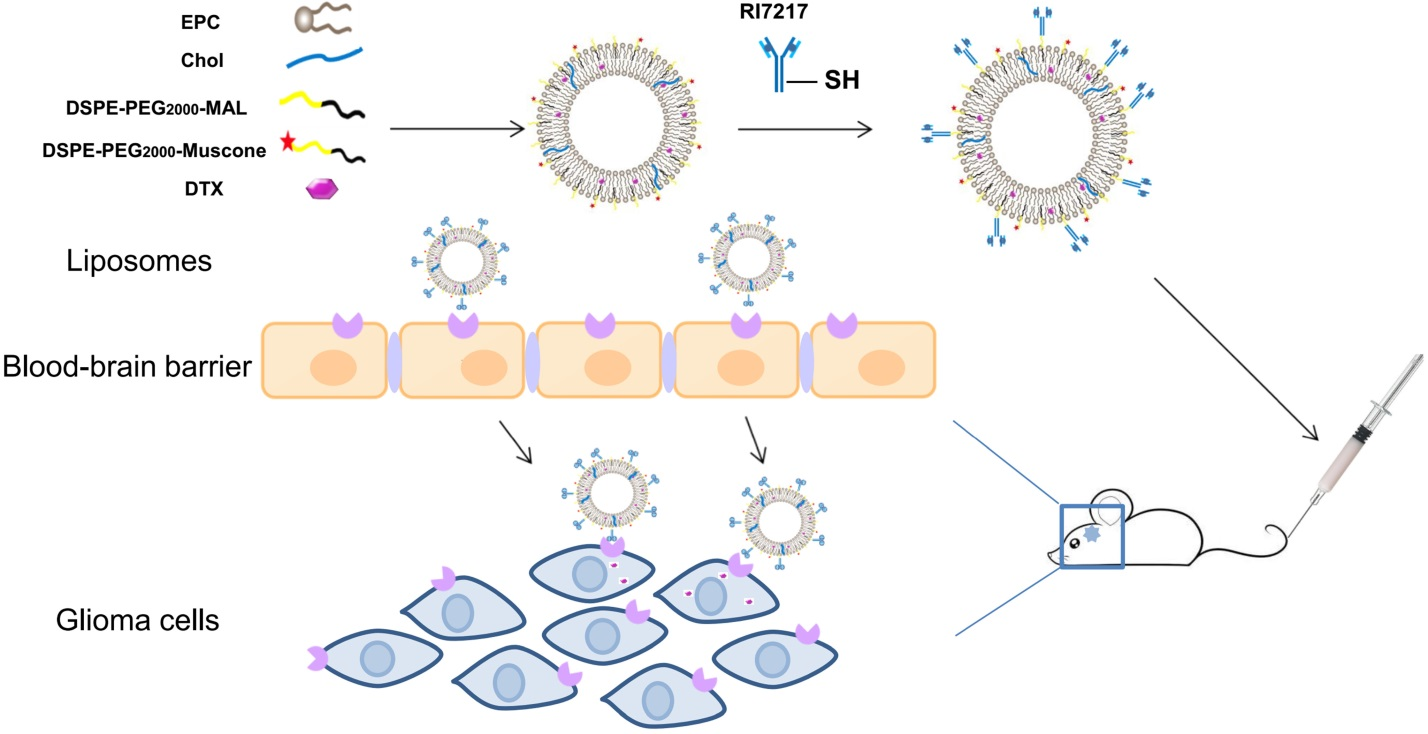
Illustration of the fabrication and BBB-penetrating and targeting glioma properties of the RI7217 and muscone co-modified DTX long-circulating liposomes for anti-glioma treatment.

The antineoplastic DTX has been reported to be effective for the treatment of breast, ovarian, non- small cell lung and brain cancers 22, 23. Sampath et al. 24 reported that DTX showed significant activity against gliomas when delivered locally 24 and hence, in this current study, DTX was chosen as a model drug for drug targeting. The effects of TCM aromatic resuscitation and modern receptor-targeted technology using constructed muscone/RI7217 co-modified DTX long-circulating liposomes were combined to increase the drug concentration in brain and enhance DTX anti-glioma effect. In this study, RI7217 and muscone co-modified DTX long- circulating liposomes were first prepared and characterized (Scheme 1). The uptake capacity and mechanism of the dual-targeted liposomes were evaluated using hCMEC/D3 cells and U87-MG glioma cells *in vitro*. The BBB penetration ability of the dual-targeted liposomes was then evaluated using *in vitro* BBB model of the hCMEC/D3 cells. A tumor spheroid model was used to investigate drug penetration into tumor tissue and the inhibitory effects on tumor growth for dual-targeted liposomes *in vitro*. Finally, the tumor targeting efficiency and *in vivo* anti-tumor efficacy of the dual-targeted liposomes were evaluated.

## Materials and Methods

### Materials

DTX was purchased from Wuhan XinxinJiali Biological Technology (Wuhan, China). Coumarin-6 (C6, purity > 98%; Sigma-Aldrich, St. Louis, USA), cholesterol (Xingzhi Chemical Factory, Shanghai, China), egg phosphatidylcholine (EPC; Lipoid GmbH, Ludwigshafen, Germany), DSPE-PEG2000 and DSPE-PEG2000-MAL (Ponsure, Shanghai, China) were sourced and used as supplied. Traut's reagent (2-iminothiohne) was purchased from Thermo Fisher Scientific (San Jose, CA, USA). Sephadex G-50, Sepharose CL-4B, trypsin, 3-(4,5-dimethylthiazol-2-yl)-2,5- diphenyltetrazolium bromide (MTT) and TritonX-100 were purchased from Solarbio Technology (Beijing, China). Hoechst 33342, rhodaminephalloidin and DiR dyes were purchased from Yeasen Biotechnology (Shanghai, China). Dulbecco's modified Eagle's medium (DMEM; Gibco, Thermo Fisher Scientific, San Jose, CA, USA), fetal bovine serum (FBS; GeminiBio, West Sacramento, CA, USA) and high-performance liquid chromatography (HPLC) solvents (Xiqiao Chemical, Shantou, China) were among the chemicals purchased and used as acquired.

U87-MG glioma cells and immortalized hCMEC/D_3_ were purchased from Guandao Biological Engineering (Shanghai, China) and when used, cultured in DMEM medium, supplemented with 10% FBS and 1% antibiotics (penicillin and streptomycin) under 5% CO_2_ at 37ºC.

### Synthesis of DSPE-PEG_2000_-Muscone

The DSPE-PEG_2000_-Muscone was synthesized according to the method proposed by Abdolahpour et al. 25. Amino-muscone, DSPE-PEG_2000_-NHS and triethylamine (in molar ratio 3:2:4) were dissolved in chloroform-methyl alcohol (2:1, v/v) and stirred at 30℃ overnight. The mixture was then washed with water (50 mL), then brine (50 mL) and dried using anhydrous Na_2_SO_4_ prior to filtration. The filtrate was subsequently evaporated to dryness and the residue was purified by silica gel chromatography by eluting with ethyl acetate-petroleum (10:1, v/v) to produce a yellow oily product after rotary evaporation. The product was identified using matrix-assisted laser desorption/ionization time of flight mass spectrometry (MALDI-TOF-MS, Shimadzu, Kyoto, Japan) and nuclear magnetic resonance spectroscopy (400 MHz 1H NMR, AVANCE III 400, Bruker, Switzerland).

### Preparation of liposomes

Liposomes (LP) were prepared by thin film hydration using PEG-LP, consisting of EPC:Chol: DSPE-PEG2000 (100:20:5, mole ratio), and M-LP, consisting of EPC:Chol:DSPE-PEG2000:DSPE-PEG2000-muscone (100:20:5:0.1, mole ratio) 26. DTX was added at a drug to lipid ratio of 1:30. The liposomes prepared and DTX-lipid mixture were dissolved in CHCl_3_ and solvent subsequently removed by rotary evaporation. After vacuum drying over 2 h, the lipid membrane was redissolved in 5 mL PBS (0.01 M, pH 7.4) over 30 min and size was reduced by probe sonication for 3 min. For fluorescently labeled liposomes, coumarin 6 or DiR and lipid were added to chloroform at a dye to lipid ratio of 1:30.

PEG-LP was used to prepare ligand-modified liposomes (RI-LP) which consisted of EPC:Chol: DSPE-PEG2000:DSPE-PEG2000-MAL in the mole ratio of 100:20:5:1. The preparation of RI-LP was carried out by thiolation of RI7217 by the reaction with Traut's reagent (1:40 n/n) in 0.15 M Na borate buffer at pH 8.5 27-29. An EDTA solution (0.01 mM) used to sequester divalent metal ions in the solution. The mixture was stirred at room temperature for 2 h and the excess Traut's reagent removed using a Sephadex G50 column (20×1 cm) 30. Thiolated RI7217 was then incubated with PEG-LP for 4 h at a molar ratio RI7217:DSPE-PEG_2000_-MAL of 1:10 31 and overnight at 4°C. RI-LP was separated from free RI7217 by gel filtration chromatography (Sepharose CL-4B, 25×1.5 cm) using phosphate buffer as the eluent 32. The concentration of liposomes was determined by quantifying phospholipids using the Stewart method 33. The amount of RI7217 labeled on the surface of liposomes was determined by Bradford assay 32, 34. The preparation of RI-LP-M was consistent with that of RI-LP with the PEG-LP replaced by M-LP.

### Characterization of liposomes

The particle size, polydispersity index (PDI) and zeta potential of PEG-LP, M-LP, RI-LP and RI-LP-M were determined using the Zetasizer Nano ZS (Malvern Instruments, Worcestershire, UK).

The morphology of PEG-LP, M-LP, RI-LP and RI-LP-M was observed using a transmission electron microscope (TEM; Jeol, Tokyo, Japan). The sample was mixed with 1% phosphotungstic acid (1:1, v/v) then dripped onto the carbon film of a copper TEM grid, reacted for 2 min and air-dried at room temperature 35.

Encapsulation efficiency (EE) and drug loading capacity (DLC) were determined according to the equations, EE (%) = (actual DTX/total DTX)×100 and DLC (%) = (actual DTX/total liposomes)x100, respectively. Where actual DTX was the encapsulated DTX concentration, total DTX was the concentration of DTX in the liposome suspension. Liposomes were dialyzed using a 10kD dialysis bag for 8 h to remove free DTX 36. High performance liquid chromatography (HPLC; LC-20AT, Shimadzu, Kyoto, Japan) analysis of DTX was performed after liposomes disruption using methanol. Chromatographic separation was by a Hypersil BDS C18 column (250 mm × 4.6 mm, 5 μm; Thermo Fisher Scientific, San Jose, CA, USA) 37 at 28ºC using a mobile phase of water:acetonitrile (45:55, v/v) 38 at 1.0 mL/min. An injection volume of 10 µL was made and DTX detected at 230 nm with a UV/Vis detector.

The *in vitro* stability of formulations based on size and turbidity were evaluated in fetal bovine serum (FBS) 39,40 by mixing equal volumes of liposomes and FBS at 37 °C with oscillation at 30 rpm. At predetermined time points (1, 2, 4, 8, 12 and 24 h), 200 μL aliquot was withdrawn and deposited into a 96-well plate to measure the transmittance at 750 nm by a microplate reader (Infinite M200 PRO, Tecan Austria GmbH, Grodig, Austria) and another 200 μL aliquot was used for dynamic and electrophoretic light scattering (Zetasizer Nano ZS, Malvern Instruments, Worcestershire, UK) to determine size and zeta potential, respectively.

*In vitro* release of the formulations was carried out using the dialysis method 39. A 2 mL aliquot was introduced into a dialysis bag (MWCO, 8000-12000 Da), sealed and immersed into 30 mL PBS (pH 7.4) containing sodium salicylate (1 mol·L^-1^) at 37°C with stirring at 100 rpm. Release was carried out to different time points, for up to 72 h. For each time point, the sample in the bag was removed and extracted with chloroform for 3 times, 2 mL each time, cumulated chloroform washings was dried using nitrogen, redissolved in 1 mL methanol, filtered through 0.22 μm and 20 μL injected for DTX determination by HPLC as described previously.

### Cytotoxicity assay

Cytotoxicity of free DTX and the various formulations against U87-MG cells was determined by the MTT assay 41. Cells were seeded in 96-well plate at a density of 1× 10^4^ cells/well until grown to 80% confluence. The media were then replaced and free DTX or different formulations of serial DTX concentrations were introduced to the cells and incubated at 37°C and 5% CO_2_ for 24 h. The free DTX was the positive control, complete medium as negative control and wells without cells were set as the background. After 24 h, the medium was removed and the cells were washed with PBS. Fresh medium (without serum) containing 0.5 mg/mL of MTT was added. After cells were incubated at 37°C and 5% CO_2_ for 4 h, the medium was replaced by DMSO. The value of optical density (OD) was measured at 570 nm using a grating type continuous wavelength microplate reader (Infinite M200 PRO, Tecan Austria GmbH, Grodig, Austria) to analyze cell viability. Live cells (%) = (A_570_ sample - A_570_ background) / (A_570_ control - A_570_ background) ×100 42, where the A_570_ control is the OD_570_ value of the negative control group and A_570_ background is the background group OD_570_ value.

### The uptake of liposomes

For quantitative research, C6 was substituted for DTX to prepare the liposomes. It provided the possibility for fluorescence detection while avoiding DTX cytotoxicity. U87 and hCMEC/D3 cells were seeded in 6-well plates at a density of 5×10^5^ cells/well and incubated at 37°C and 5% CO_2_. After 24 h, the cells were incubated with C6 loaded liposomes (PEG-LP-C6, M-LP-C6, RI-LP-C6, RI-LP-M-C6, RI-LP-C6+M) at 37°C and 5% CO_2_ 43. The concentration of C6 was 10 μM and for the addition of 0.4 μg/mL muscone in RI-LP-C6+M group. After 4 h, the medium was removed, cells washed thrice with PBS then collected and suspended in PBS. The cell samples were analyzed by flow cytometry (FACS AriaⅢ, BD Biosciences, Franklin Lakes, NJ, USA).

For qualitative studies, U87 and hCMEC/D3 cells were seeded on glass cover slips in 24-well plate at a density of 1×10^4^ cells/well. The cells were incubated for 24 h at 37°C and 5% CO_2_ and then incubated with C6 loaded liposomes at 37 °C and 5% CO_2_ for 4 h. The cells were washed 3 times with cold PBS and fixed with 4% paraformaldehyde for 20 min at room temperature. Next, the cells were washed thrice with PBS. The cells were permeabilized with 0.5% Triton-X100 in PBS for 5 min and then blocked with goat serum for 30 min 44. The cells were washed thrice with PBS and incubated with 5 μg/ml Hochest 33342 for 10 min to stain the nuclei 45, 46. The cells were washed thrice with PBS, slides air-dried and mounted for observation using a fluorescence microscope (DM500, Zeiss, Germany).

The influence of various inhibitors on cellular uptake of liposomes was studied using hCMEC/D3 cells, pre-incubated with various inhibitors, namely colchicine (5μg/mL) 47, amiloride (15μg/mL) 48, chlorpromazine (10 μg/mL) 49 and filipin (5 μg/mL) 50, for 30 min at 37°C. The medium containing the inhibitor was then discarded and fresh medium containing C6 loaded liposomes was added, then cultured at 37°C for 4 h. The cells were washed thrice with PBS and lysed by 1% Triton X-100 at 4°C for more than 30 min in the dark. The fluorescence intensity was determined using a filter-type multi-function microplate reader (Infinite F500, Tecan Austria GmbH, Grodig, Austria).

### The penetrating ability and inhibitory effect to tumor spheroids

As described previously, three-dimensional tumor spheroids were prepared 51, 52. Agarose solution (2%, w/v) was prepared in serum-free DMEM. U87-MG cells were seeded at 5×10^3^ cells/well in 96-well plates pre-plated with low-melting agarose, shaken for 5 min and incubated at 37°C in the presence of 5% CO_2_. The spheroids of approximately 400 µm in diameter were chosen for incubation with C6 loaded formulations (PEG-LP-C6, M-LP-C6, RI-LP-C6, RI-LP-M-C6, RI-LP-C6+M) for 4 h at 37°C 53. The concentration of C6 was 10 μM with an addition of 0.4 μg/mL muscone in the RI-LP-C6+M group. After incubation, the spheroids were washed with cold PBS and viewed under confocal fluorescence microscope to quantify the penetrating capacity of the formulations by scanning different layers, from the top to middle of the spheroids.

Assessment of growth inhibition by liposomes against U87-MG glioma spheroids was evaluated by culturing the spheroids in serum-free DMEM, free DTX solution, PEG-LP-DTX, M-LP-DTX, RI-LP-DTX, RI-LP-M-DTX at 37°C under 5% CO_2_. The final concentration of DTX used was 35 μg/mL. The culture medium was changed every 2 days. Growth inhibition was monitored daily by measuring the size of the spheroids with an inverted phase microscope. The major (d_max_) and minor (d_min_) diameters of each spheroid was measured and spheroid volume calculated using the following formula, V=(π×d_max_×d_min_)/6. The glioma spheroid volume ratio was estimated from the formula, R=(V_day i_/V_day 0_)×100% where V_day i_ is the U87 glioma spheroid volume at the ith day after administration of the drug and V_day 0_ is the U87 glioma spheroid volume before administration 54. The data obtained is expressed as mean ± SD (n = 3).

### Endothelial permeability test of hCMEC/D3 cells

The hCMEC/D3 cells were seeded at 5×10^4^ cells/well on Type I collagen precoated Transwell filters (polycarbonate, 12 wells, 0.4 μm pore size). The medium was replaced every 2 days. Transendothelial electrical resistance (TEER; Millicell ERS-2, Millipore, USA) measurement was used to verify cell barrier integrity. After 12-15 days, only cells showing TEER value between 65 and 89 Ω⋅cm^2^ 55 were used for the transport assay study.

The quality of the monolayer was further evaluated by determining the lucifier yellow permeability and results obtained were compared with previously reported values 32 to ensure that the liposomes did not disrupt the cell membrane barrier.

A sample of C6 loaded liposomes was placed on the upper chamber (200 nmol lipid/well) and the fluorescence intensity of the sample in the upper and lower chambers was measured at 15, 30, 60 and 120 min in phenol red-free DMEM at λex = 485 nm and λem = 528 nm using a filter type multi-function microplate reader (Infinite **®**F500, Tecan Austria GmbH, Grodig, Austria). The apparent permeability values across the blank Transwell inserts were carried out in triplicates with no cells inoculated.

### *In vivo* fluorescent imaging

In order to observe *in vivo* the real-time distribution of liposomes, DiR-loaded liposomes were prepared for glioma-targeting evaluation using BALB/c nude mice which were anesthetized and fixed into a stereotactic device. The orthotopic U87-MG glioma bearing mice were established by injecting 5 μL U87-MG cell suspension (1×10^5^ cells/mL) into the right brain (striatum, 1.8 mm right lateral to the bregma and 3 mm of depth) of the mice 54. On the 8th day after tumor implantation, 3 nude mice were randomly selected and brains were sampled for haematoxylin and eosin (H&E) staining. In addition, 200 μL of DiR-loaded liposomes (PEG-LP-DiR, M-LP-DiR, RI-LP-DiR, RI-LP-M-DiR and RI-LP-DiR+M) were injected into the tail vein of mice bearing U87-MG glioma. Muscone and DiR were administered at a dose of 1.0 mg/kg. The nude mice were scanned at 2, 4, 8, 12 and 24 h after administration using whole-body fluorescent imaging system. At 24 h after administration, the nude mice were sacrificed and organs, brain, heart, liver, spleen, lung and kidney, were removed for further fluorescent imaging (Ex=740 nm/Em=790 nm).

### *In vivo* survival monitoring

The U87-MG glioma bearing BALB/c nude mice was established as described previously. On day 9 post-implantation, the nude mice were randomly divided into 5 groups, each 10 mice: saline group (blank control), PEG-LP-DTX group, M-LP-DTX group, RI-LP-DTX group, RI-LP-M-DTX group and RI-LP-DTX+M (gavage) group. The mice received intravenous infusions with doses equivalent to DTX of 5 mg/kg, repeating after every 3 days for 3 additional doses. Survival time of mice all groups was recorded and the Kaplan-Meier survival curve plotted for each group.

## Results and Discussion

### Characterization of DSPE-PEG_2000_-Muscone

**Figure 1:**
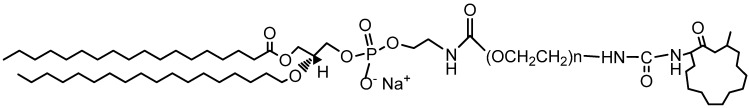
Structure of DSPE-PEG_2000_-Muscone.

DSPE-PEG_2000_-Muscone was synthesized (Supplementary Figure S1A) and complex formed confirmed using MALDI-TOF- MS (Supplementary Figure S1B) together with ^1^H NMR (Supplementary Figure S1C). The MALDI-TOF-MS spectrum indicated an average mass of the conjugate to be m/z 2257 while the molecular weight of DSPE-PEG_2000_-Muscone is 3005. The difference in masses of DSPE-PEG_2000_- Muscone and its conjugate can be explained by the loss of DSPE (MW, 748) in the conjugate. According to ^1^H NMR spectrum, the peaks of amino-protons were missing, thus confirming the formation of DSPE-PEG_2000_-Muscone (Supplementary Figure S1C). The attribution and analysis of functional groups by ^1^H-NMR was showed that the peaks in DSPE-PEG_2000_-Muscone at 0.86 and 0.90 ppm were the methyl groups (‑CH_3_). Additionally, the proton peaks at 1.20∼1.34 ppm were methylene (-CH_2_-). Besides, proton peaks at 3.46 ppm signified the protons of PEG_2000_ ethyleneoxy groups (-CH_2_CH_2_O-). Based on the above, DSPE-PEG_2000_-Muscone was successfully fabricated and structure of DSPE-PEG_2000_-Muscone resolved (Figure 1). The purity of the material was 95%.

### Preparation and characterization of LP

**Figure 2:**
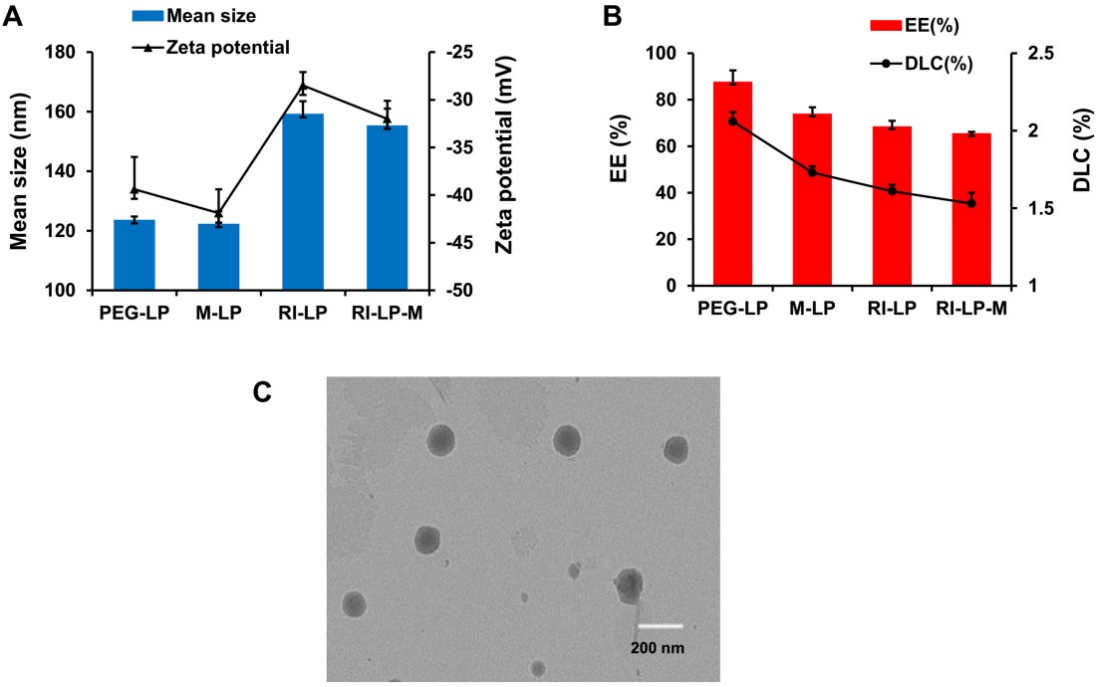
(A) Particle size and zeta potential of the liposomes groups. (B) EE and DLC of the liposomes groups (n=3, mean±SD). (C) TEM of RI-LP-M. (bar represents 200 nm).

Liposomes were prepared by the thin-film hydration method as described earlier and characterized for size, PDI, zeta potential, TEM and RI7217 coupling efficiency. PEG-LP had a mean particle size of 123.5±1.3 nm with a zeta potential of -39.4±3.4 mV. M-LP showed rather similar values, mean size of 122.2 ± 4.4 nm and zeta potential of -41.9±2.5 mV (Figure 2A). The mean sizes of RI7217 modified liposomes, RI-LP and RI-LP-M were 159.1 ± 4.4 and 155.2±5.8 nm, respectively, showing some increase in size due to the coupling of ligand to the surface of the liposomes 56. For zeta potential, RI-LP and RI-LP-M showed a small decrease in absolute values, -28.5±1.4 and -32.0±1.9 mV, respectively, (Figure 2A) since the RI7217 functional group with the positive charge modified on the surface of liposomes 57. In all cases, the values of PDI were less than 0.3, indicating that the liposomes batches were homogeneous in size. The liposomes were observed using a TEM after negative staining with phosphotungstic acid and the spherical RI-LP-M particles as seen in Figure 2C has an average particle size of about 150 nm, which were consistent with sizing by dynamic laser scattering. The morphology of PEG-LP, M-LP and RI-LP are globose, similar to that of RI-LP-M but micrographs were not shown. The amount of RI7217 coupled with liposomes was calculated based on the concept that 100 nM liposome contains ∼100,000 molecules of phospholipids 58. The results indicated that RI7217, DSPE-PEG_2000_-MAL and EPC in molar ratio of 1:10:1000 with the conjugation efficiency of RI7217 at 52.68% had an average number of RI7217 moieties attached to the surface of each liposome at about 30.

PEG-LP-DTX, M-LP-DTX, RI-LP-DTX and RI-LP-M-DTX had EE of 87.56±5.05%, 73.80±2.93%, 68.33±2.59% and 65.37±0.78%, respectively, while for DLC, values obtained were 2.06 ± 0.06%, 1.73±0.04%, 1.61±0.04% and 1.53±0.07%, respectively (Figure 2B). Comparing PEG-LP-DTX and M-LP-DTX, the drug EE of RI7217 antibodies modified liposomes decreased. This might be due the liposomes modifying RI7217 antibodies, requiring the use of agarose gel column CL-4B to remove uncoupled antibodies. It is speculated that some liposomes maybe impaired by the gel in the process which led to some drug leakage causing a decrease in EE of RI7217 antibodies modified liposomes.

**Figure 3:**
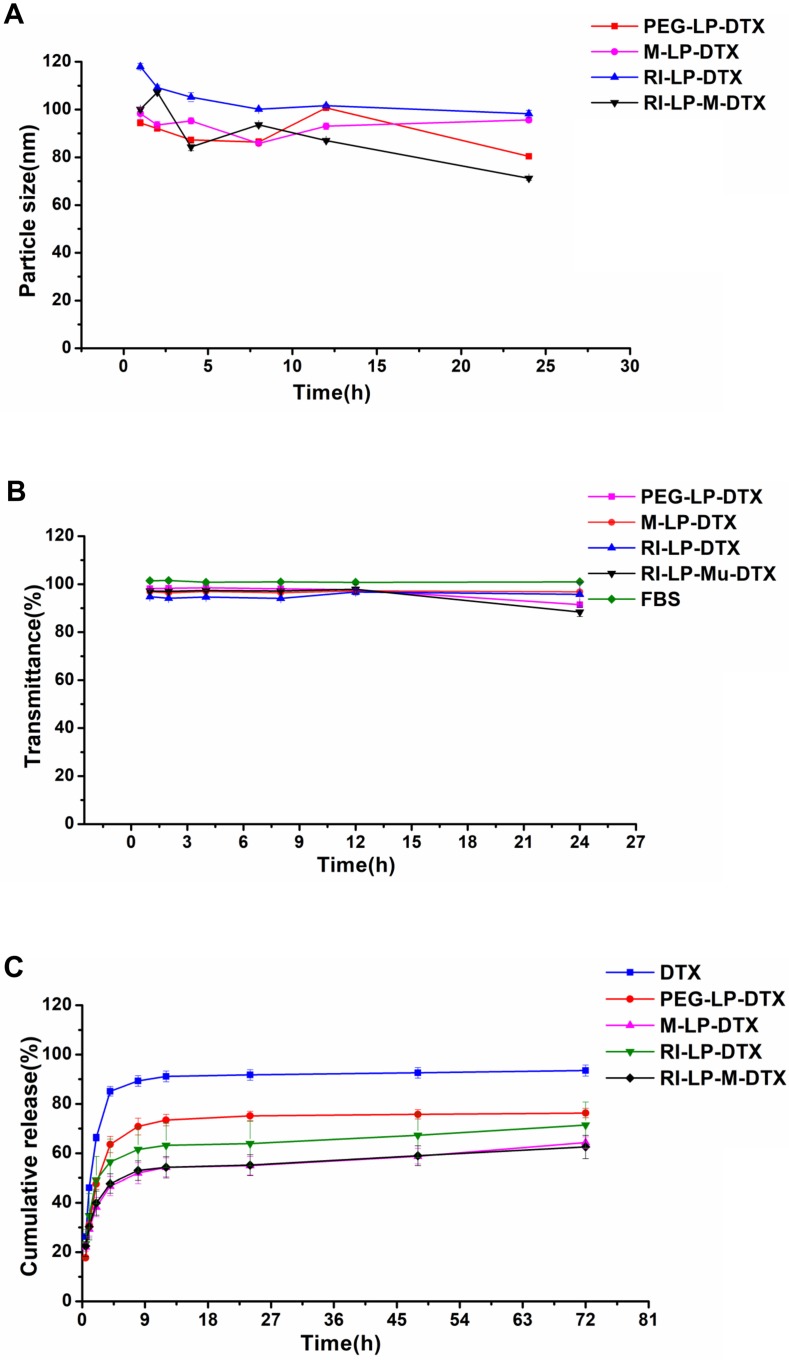
(A) The size of formulations in 50% FBS over time. (B) The variation in turbidity (represented by transmittance) of formulations in 50% FBS (n=3, mean ±SD). (C) The *in vitro* release profiles of formulations in PBS (n=3, mean ±SD).

The *in vitro* stability results of formulations are shown in Figures 3A and 3B. The particle size and transmittance of all the formulations were relatively stable over 24 h, suggesting that for all of the formulations, no aggregation occurred in the presence of serum. Hence, these results found all the formulations stable over 24 h and should be suitable for use by the intravenous injection route.

The *in vitro* release profiles of formulations are shown in Figure 3C. DTX was rapidly released from DTX solution but PEG-LP-DTX showed a significant sustained DTX release profile. The release profile of RI-LP-DTX was much slower than that of the PEG-LP-DTX which indicated the retention of RI7217 modified DTX liposomes in the dialysis bag had prolonged the DTX release. Similarly, M-LP-DTX also exhibited more significant sustained DTX release properties. It was proposed that electrostatic interaction between muscone and DTX may had hindered the release of DTX. The release curve from RI-LP-M-DTX exhibited similar sustained release of DTX, possibly the result of the combination of the retention of RI7217 in the dialysis bag and electrostatic interaction between muscone and DTX.

### Cytotoxicity assay

**Figure 4:**
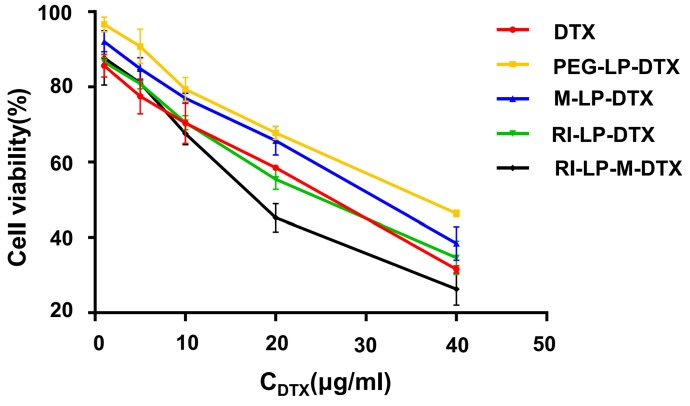
*In vitro* cytotoxicity of DTX loaded liposomes on U87-MG cells (n=3, mean ±SD).

The *in vitro* cell cytotoxicity of free DTX and DTX loaded liposomes were investigated on U87-MG cells at DTX concentrations of 5, 10, 20, 30, 40 and 50 μg/mL by MTT assay. The cytotoxicity results are shown in Figure 4. The inhibition behavior of free DTX and DTX loaded liposomes on U87-MG cells exhibited concentration-dependent results. After incubation with U87-MG cells for 24 h, IC50 values of DTX, PEG-LP-DTX, M-LP-DTX, RI-LP-DTX and RI-LP-M-DTX were calculated from the cytotoxicity results of 25.0, 43.8, 36.1, 25.9 and 19.2 μg/ mL, respectively. It was found that free DTX exhibited stronger inhibition than PEG-LP-DTX which inferred that the free drug was quickly absorbed by passive diffusion into cells under *in vitro* conditions and rapidly affected cell growth without any drug release processes 39,59. RI‑LP‑DTX and RI-LP-M-DTX showed higher cytotoxicity than PEG-LP-DTX or M‑LP-DTX. The above results indicated that RI7217 antibody modification enhanced the anti-proliferative effect which possibly was related to enhanced cellular uptake of the formulations. The cytotoxicity results confirmed that muscone and RI7217 co-modification facilitated cellular internalization of liposomes and enhanced the inhibitory effect of chemotherapeutic drugs on U87-MG glioma cells 60**.**

### *In vitro* cellular uptake evaluation

Cell uptake of the different ligand-modified liposomes was investigated using hCMEC/D3 and U87-MG cells by determining the extend of uptake qualitatively and quantitatively. The coumarin-6 (C6) loaded liposomes (PEG-LP-C6, M-LP-C6, RI-LP-C6, RI-LP-M-C6, RI-LP-C6+M) were incubated for 4 h with hCMEC/D3 and U87-MG cells and uptake levels were evaluated using fluorescence microscopy and flow cytometry.

**Figure 5:**
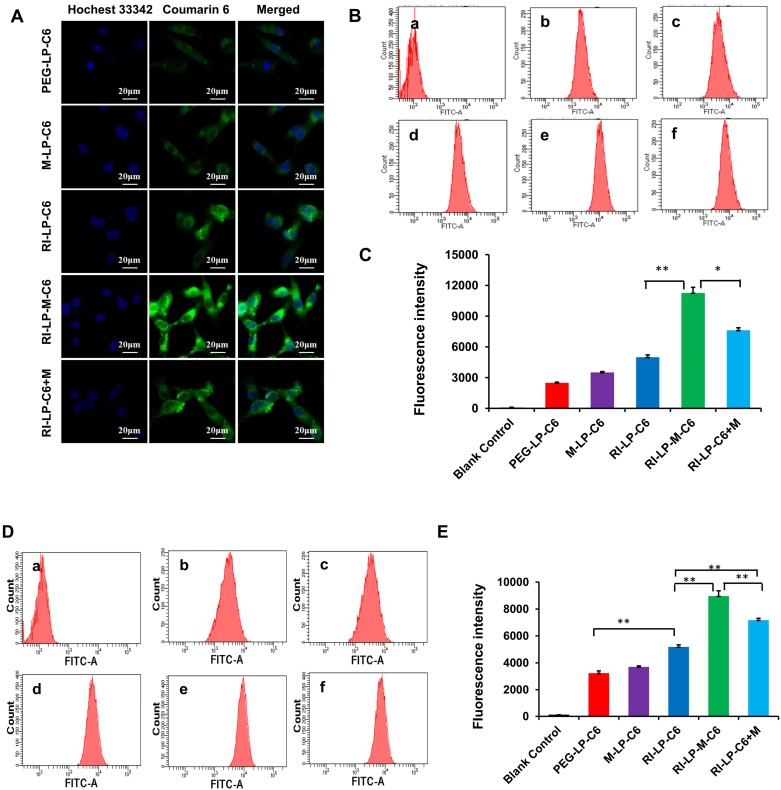
Cellular uptake of C6 loaded liposomes for 4 h. (A) Fluorescence microscope images of U87-MG cells treated with different C6 loaded liposomes. (B) Intracellular fluorescence intensity of U87-MG cells analyzed by flow cytometry after treated with different C6 loaded formulations. (C) The mean fluorescence intensity values of C6 loaded formulations in U87-MG Cells were semi-quantitatively analyzed. (D) Intracellular fluorescence intensity of hCMEC/D3 cells analyzed by flow cytometry after treated with different C6 loaded formulations. (E) The mean fluorescence intensity values of C6 loaded formulations in hCMEC/D3 Cells were semi-quantitatively analyzed.

For qualitative uptake, fluorescence microscope images of C6 loaded liposomes were taken after incubation with U87-MG cells at 37°C for 4 h. In Figure 5A, Hoechst 33342 which labeled the cell nucleus emitted a blue fluorescence, C6 loaded liposomes emitted green fluorescence and a merged view of the two channels were also presented. The images showed that the fluorescence intensity of the formulations group was in the following order: RI-LP-M-C6 group > RI-LP-C6 group ≈ RI-LP-C6+M group > M-LP-C6 group > PEG-LP-C6 group.

Based on the quantitative uptake findings, results of C6 loaded formulations in U87-MG cells using flow cytometry (Figures 5B and 5C) indicated that the fluorescence intensity of blank control group (free C6) was very low as free C6 was barely taken up by the U87-MG cells. The fluorescence intensity of the RI-LP-M-C6 group was highest and next, the RI-LP-C6+M group and they were 2.3x and 1.5x higher than the RI-LP-C6 group, respectively. In contrast, the fluorescence intensity of the RI-LP-C6 group was twice that of the PEG-LP-C6 group and that of the M-LP-C6 group only slightly higher than the PEG-LP-C6 group (p>0.05).

The quantitative uptake results of C6 loaded formulations in hCMEC/D3 cells using flow cytometry are shown in Figures 5D and 5E. The uptake trend of the formulation groups in hCMEC/D3 cells mirrored the uptake in the U87-MG cells albeit the results were generally more distinctive, often with p > 0.01.

The findings on cellular uptake had indicated that RI7217 antibody modification promoted the uptake of liposomes. It was reasonable to assume that the RI7217 antibody was internalized by specifically recognizing TfR through a receptor-mediated pathway on the surface of glioma cells and cerebral vascular endothelial cells 61. However, compared with the PEG-LP-C6 group, muscone mono-modified liposomes (M-LP-C6) did not significantly increase uptake by U87-MG and hCMEC/D3 cells. Compared with the RI-LP-C6 group, RI7217 antibody modified liposomes and the co-incubation with muscone (RI-LP-C6+M) enhanced cellular uptake, which was consistent with the action by borneol as reported in literature 62. Meanwhile, muscone and RI7217 co-modification was also most effective on promoting cellular uptake, indicating synergy between muscone and RI7217.

**Figure 6:**
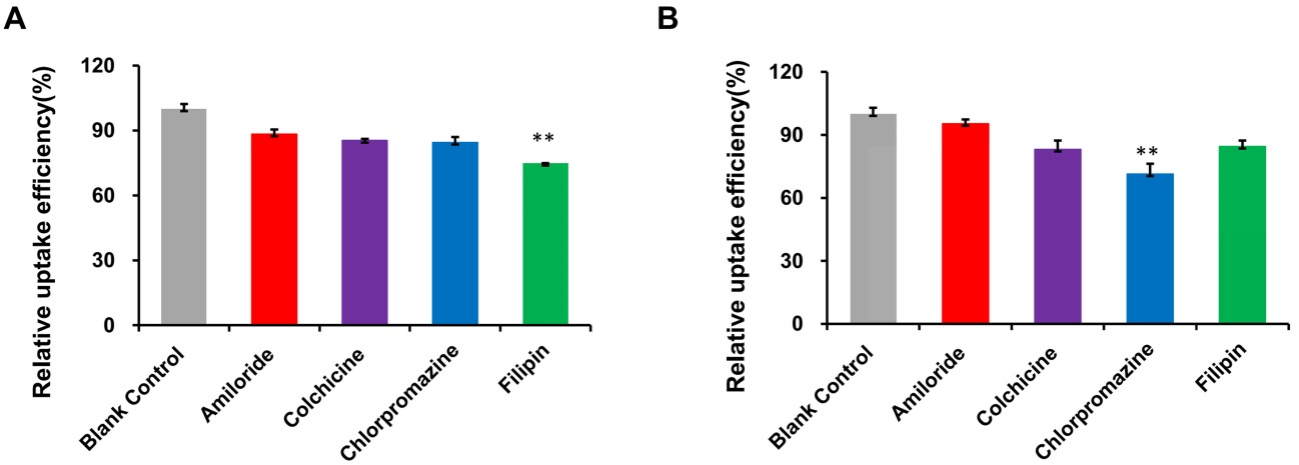
(A) Uptake mechanism of RI-LP-M in U87-MG cells. (B) Uptake mechanism of RI-LP-M in hCMEC/D3 cells.

The mechanism of RI-LP-M uptake was further investigated by examining the extent of uptake inhibition of RI-LP-M by U87-MG cells by various inhibitors (Figure 6A). Compared to the control, the fluorescence intensity decreased by 11.6, 14.6, 15.5 and 25.2% after treatment with amiloride, colchicine, chlorpromazine and filipin, respectively. Fluorescence intensity decreased significantly after pre-incubation with the clathrin inhibitor chlorpromazine (p<0.01), the caveolin inhibitor filipin (p<0.01). This finding indicated that clathrin and caveolin mediated endocytosis were involved in the uptake process of RI-LP-M by U87-MG cells. Clathrin and caveolin mediated endocytosis had been reported to be cholesterol dependent 63 which suggested that the uptake of RI-LP-M by U87-MG cells might had undergone a cholesterol dependent uptake mechanism but this proposal required further validation.

Results for RI-LP-M uptake inhibition into hCMEC/D3 cells are shown in Figure 6B. Compared with the control group, the uptake of RI-LP-M was partially inhibited by amiloride, colchicine, chlorpromazine and filipin by 4.5, 16.9, 28.6 and 15.4%, respectively. Fluorescence intensity decreased significantly after pre-incubation of the clathrin inhibitor chlorpromazine (p< 0.01), indicating that clathrin mediated endocytosis was more strongly involved. Thus, the uptake mechanism of muscone and RI7217 co-modified liposomes by hCMEC/D3 cells had somewhat differed from that by the U87-MG cells.

### Tumor spheroid penetration

**Figure 7:**
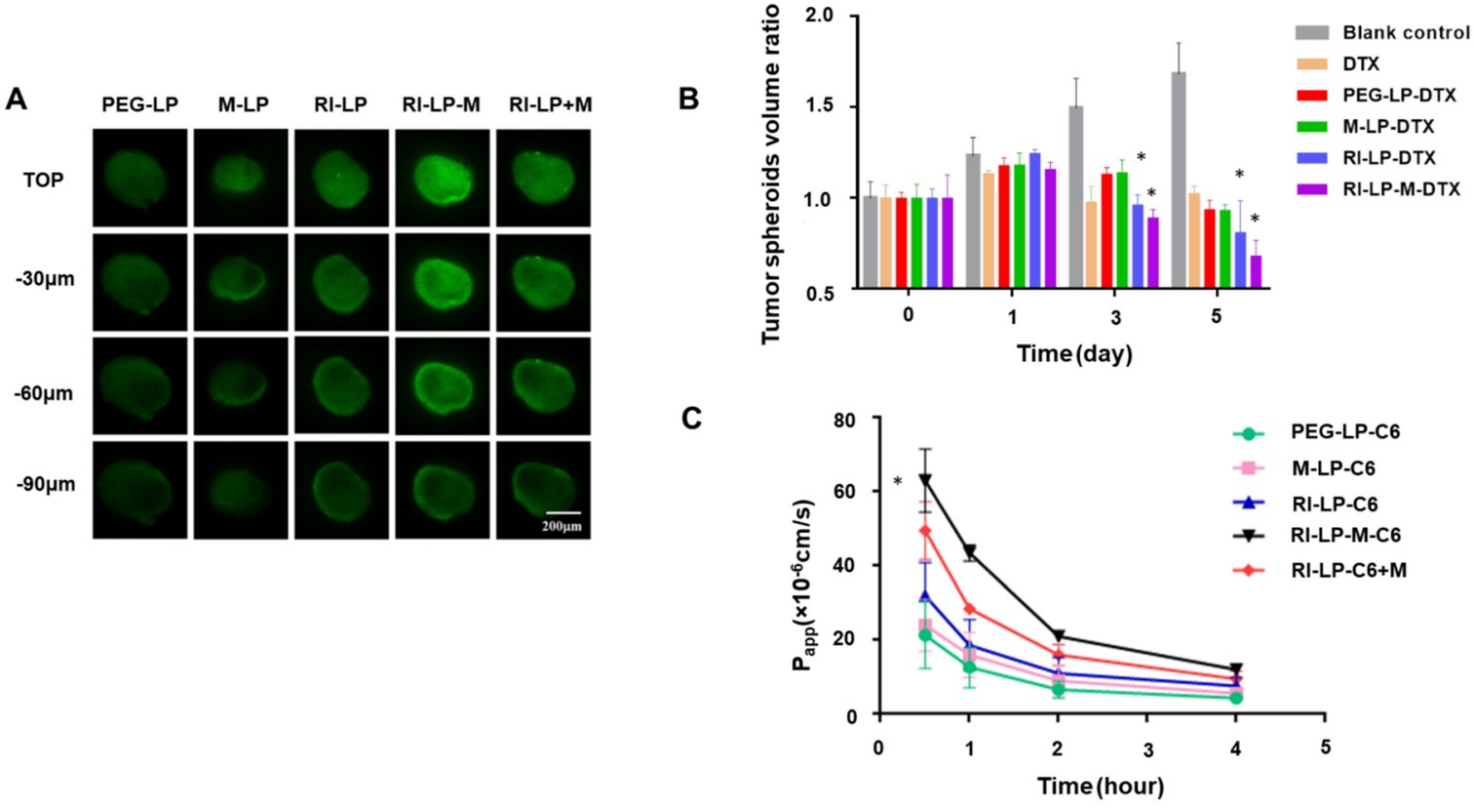
(A) Uptake of C6 loaded liposomes by U87-MG glioma spheroids for 4 h. (B) Inhibitory effect of various formulations on U87-MG glioma spheroids (n=3); * p< 0.05. (C) The P_app_ of C6 loaded liposomes through hCMEC/D3 cell monolayers at 0.5, 1, 2 and 4 h.

Due to dense cell packing, the interstitial pressure in solid tumor is often remarkably high compared to normal tissues and this could impede drug delivery into the deep tumor tissue 64-66. Thus, monolayer cells model could not accurately reflect the accumulation effect of formulations *in vivo*. The use of solid tumor spheroids as *in vitro* model could establish better congruity with the *in vivo* microenvironment of solid tumors 54, 67. After incubating with the formulations, fluorescence images were captured using CLSM from the top to middle layers of the U87‑MG tumor spheroids (Figure 7A). The tumor spheroids of PEG-LP group displayed the weakest fluorescence intensity from the outer (top) to middle layers and the M-LP group showed slightly stronger fluorescence intensity. However, compared with PEG-LP and M-LP groups, RI-LP, RI-LP-M and RI-LP+M groups exhibited considerably stronger fluorescence intensities with deeper penetration into the core of the tumor spheroids. RI-LP+M group was slightly stronger than that of RI-LP group and RI-LP-M group had the strongest fluorescence intensity. These findings had reflected the results on cellular uptake by U87-MG cells. Clearly, the results thus far had indicated that the RI7217 and muscone co-modified targeted liposomes improved the penetrating properties of formulations into the deep regions of U87-MG tumor spheroids. Furthermore, it should also be recognized that muscone modification increased the permeability of RI7217 antibody modified targeted liposomes.

### Growth inhibition of tumor spheroids

The inhibitory effect on U87-MG tumor spheroids after treatment with different formulations are displayed in Figure 7B. On day 5 post-administration, the tumor spheroids in the blank control group continued to grow unabated and tumor volume increased. In contrast, the tumor spheroid volumes administrated with DTX initially grew and then decreased. On day 5, the average relative volumes of the tumor spheroids were in the following order: RI-LP-M-DTX < RI-LP-DTX< M-LP-DTX < PEG-LP-DTX < blank control. By comparison, the groups, PEG-LP-DTX and M-LP-DTX, were minimally effective and RI-LP-DTX showed a stronger growth inhibitory effect on tumor spheroids (p<0.05). Overall, RI-LP-M-DTX had the best outcome and was even more effective than RI-LP-DTX (p<0.05), indicating that U87-MG glioma spheroids were more susceptible to RI7217/muscone co-modified liposomes. The results were consistent with the *in vitro* penetration results using U87-MG tumor spheroids. The findings also inferred that as RI7217 and muscone co-modified liposomes showed increased penetration into tumor spheroids, cell death occurred and as dead tumor cells were shed, the spheroids became smaller. The results clearly demonstrated that the use of *in vitro* tumor spheroids had accurately imitated the microenvironment of solid tumors *in vivo.* The effective tumor spheroid inhibitory effect by RI-LP-M-DTX had clearly predicted its therapeutic effect *in vivo*.

### Endothelial permeability test of hCMEC/D3 cells

*In vitro* transport of liposomes across the BBB was evaluated using Transwell cultured hCMEC/D3 cell monolayers. The permeability coefficient (P_app_) of lucifer yellow was evaluated to exclude effects of the tight junction triggered by liposomes. Lucifer yellow permeability ranged between 1.83×10^-5^~2.39×10^-5^ cm/s, in agreement with reported literature values 68 which indicated no adverse effect on cell monolayer integrity. During the penetration process of cell monolayers, high P_app_ implied the high cell permeability 32. The P_app_ values of liposomes prepared are shown in Figure 7C. Muscone mono-modified liposomes only marginally increased cell monolayer permeability as the absence of RI7217 antibody modification limited the permeability of the liposomes. In contrast, RI7217 antibody modified liposomes and the co-incubation with muscone showed higher increases in permeability across the monolayer. Thus, beyond the improved permeation effect by RI7217 antibody modification, the synergistic muscone and RI7217 co-modified liposomes showed the highest permeability, consistent with the findings of cell uptake by the formulations.

### *In vivo* fluorescence imaging

**Figure 8:**
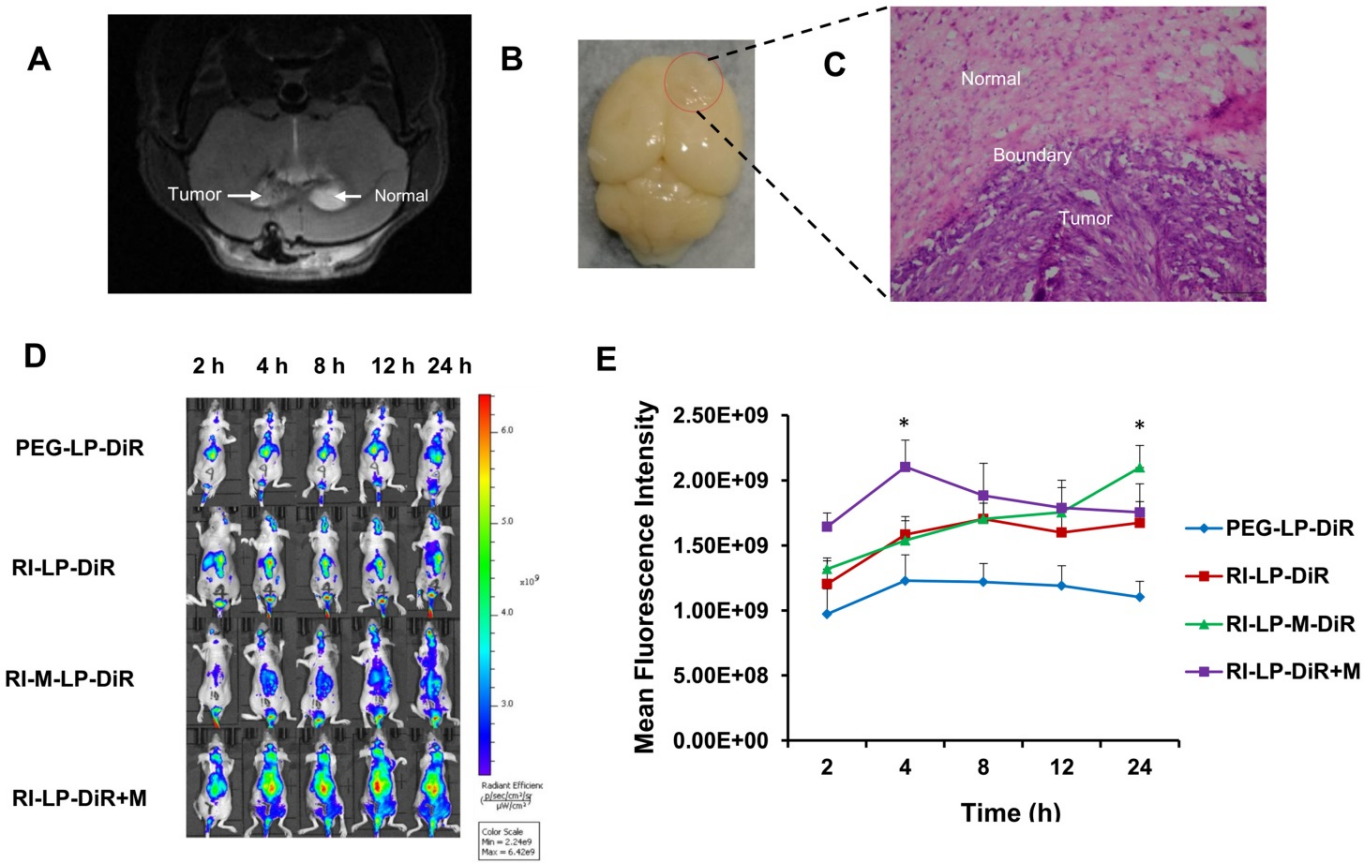
(A) The brain MRI images of glioma-bearing mouse. (B) Brain with orthotopic glioma was removed after nude mice with intracranial U87-MG glioma 8 days post-implantation. (C) Micrograph of histopathology section of brain tissue in nude mice (HE, 20×). (D) *In vivo* real-time imaging. The U87-MG orthotopic tumor-bearing nude mice were given a tail vein injection of PEG-LP-DiR, RI-LP-DiR, RI-M-LP-DiR, RI-LP-DiR+M (gavage). All mice were scanned at 2, 4, 8, 12 and 24 h. (E) The mean fluorescence intensity values of DiR loaded liposomes in full brain site were semi-quantitatively analyzed.

Glioma-targeting ability of formulations were evaluated using a near-infrared dye (DiR) loaded into the liposomes for *in vivo* imaging. Magnetic resonance imaging (MRI) is a non-invasive imaging modality that can be used to confirm brain tumor formation 69. After nude mice with intracranial U87-MG glioma on 8th day post-implantation, brain MRI image of a glioma-bearing mouse is displayed in Figure 8A. Subsequently, three mice were sacrificed and brains removed (Figure 8B) followed by H&E staining histopathological section prepared and viewed under a microscope for the boundary between tumor and normal brain tissue (Figure 8C). Hence, it was ascertained that the rat brain tumor model was successfully established. The real-time *in vivo* distribution and brain targeting of the DiR loaded formulations in the glioma-bearing nude mice are shown in Figure 8D. Compared with brain distribution of the PEG-LP-DiR group, RI‑LP-DiR group increased the accumulation of DiR at the brain tumor site. The results suggested that RI7217 antibodies had specifically recognized TfR on brain endothelium and glioma cells and had promoted RI7217 antibodies modified formulations to cross the BBB through a receptor-mediated pathway 15, attributed to the brain targeting capability of formulations. Comparing PEG-LP-DiR, RI-LP-DiR and RI-LP-DiR+M (gavage) groups, the fluorescence intensity of the RI-M-LP-DiR in the brain tumor site increased significantly with time and reached the highest value at 24 h (Figure 8E). The results indicated that the RI7217 and muscone co-modification increased the distribution of liposomes at the brain tumor site. As previously discussed, the findings could be partially attributed to the RI7217 antibodies for specifically recognizing TfR on the surface of brain endothelium and glioma cells, and along with the muscone modification to increase the BBB permeability. Furthermore, RI7217 and muscone co-modified liposomes showed focused accumulation at the glioma region and less distribution outside the glioma region in brain (Figure 8D). Thus, muscone modification did not only increased liposomes' permeability to cross the BBB but also enhanced blood-brain tumor barrier (BBTB) permeability as reported in literature 70, 71. Clearly, muscone and RI7217 antibodies co-modification had shown synergistic effects.

Furthermore, the fluorescence intensity values of the formulations at the full brain site were semi-quantitatively analyzed. During the initial stages after administration, the intracerebral fluorescence at the full brain site for the RI-LP-DiR+M (gavage) group was higher than the other groups, reaching the highest value at 4 h (Figure 8E). Later, the intracerebral fluorescence intensity at full brain site of the RI-LP-DiR+M (gavage) group decreased gradually. The results suggested that muscone (gavage) had increased BBB permeability but permeability was bi-directional 21 and thus, fluorescent residence could not be sustained. Due to characteristics of muscone (gavage) promoting BBB permeability, even if receptors mediated targeting delivery existed in the RI-LP-DiR+M (gavage) group, RI-LP-DiR entry and exit via the BBB could occur rather rapidly. At 24 h post-administration, the intracerebral fluorescence intensity values at the full brain site for RI-LP-M-DiR group, RI-LP-DiR+M (gavage) group and RI-LP-DiR group were 1.90x, 1.59x and 1.52x higher than that of the PEG-LP-DiR group, with the intracerebral fluorescence intensity of RI-LP-M-DiR group being the highest. Again, RI7217 and muscone co-modified targeted liposomes showed marked accumulation at the brain tumor site and prolonged intracerebral drug presence at the brain tumor site.

### *In vivo* anti-glioma effect

**Figure 9:**
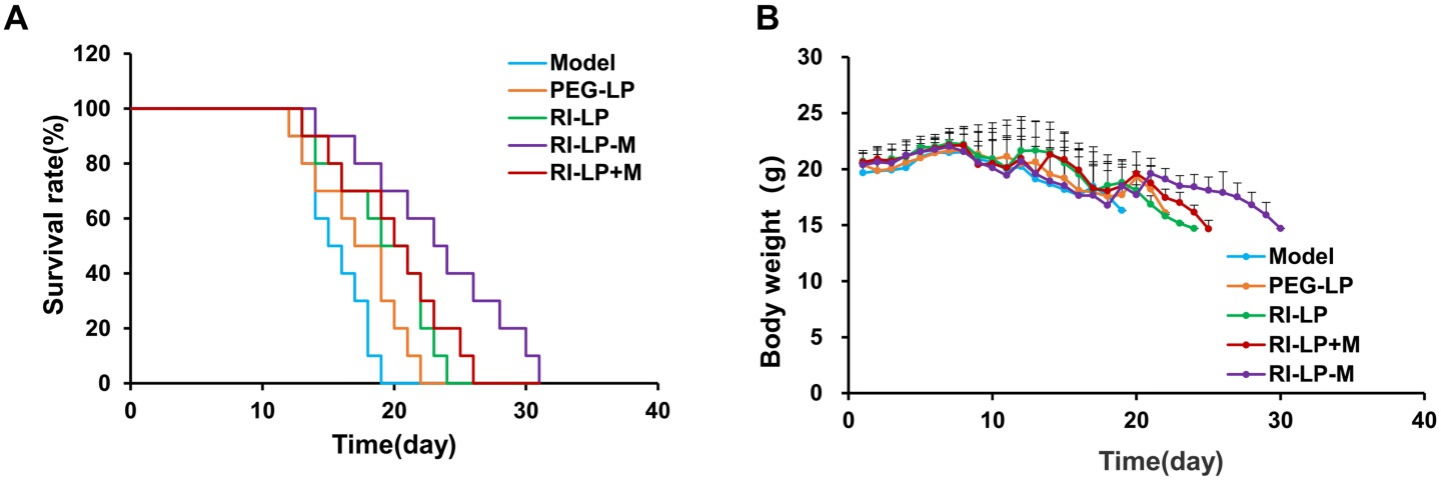
(A) Survival curves of U87-MG glioma bearing mice treated with different formulations at day 8, 11 and 14 after inoculation (each dosing, 5 mg/kg DTX). (B) Changes in body weights of U87-MG glioma-bearing mice.

The anti-glioma effects of formulations were evaluated by performing the survival curve study (Figure 9A). The median survival time of U87-MG glioma-bearing mice treated with the PEG-LP-DTX group was prolonged, from 15 to 18 days when compared with saline group. PEG-LP-DTX could passively target the glioma site via an enhanced permeability and retention (EPR) effect 5, resulting in longer median survival time by 1.2x longer than that of the saline group. The RI-LP-DTX and RI-LP-DTX+M (gavage) groups exhibited more prolonged median survival times, to 20 and 21 days, respectively, indicating that RI7217 modified liposomes had improved the therapeutic effect when compared with the PEG-LP-DTX group. Furthermore, the median survival time of the RI-LP-M-DTX group was 24 days or 1.6x longer than that of saline group. The results showed that RI7217 and muscone co-modified liposomes enhanced *in vivo* anti-glioma effect, as was visualized by the *in vivo* imaging results. Clearly, a long median survival time could be related to a high drug accumulation and retention at the glioma site in brain. In general, RI7217 and muscone co-modified liposomes showed more favorable brain targeting ability and *in vivo* anti-glioma effect than by physical addition muscone to the formulation. Thus, the addition of muscone by chemical modification allowed alternate mechanisms on altering the BBB permeability, different from that by the physical addition of muscone. However, the exact mechanism on how RI7217 and muscone co-modified liposomes enhanced the permeability of the BBB requires further investigations and is beyond this present study. Moreover, the body weight of U87-MG glioma- bearing mice in each group showed no obvious differences during experimental period (Figure 9B). There was no apparent body weight change among all the study groups which suggested that all the formulations inhibit U87-MG glioma growth *in vivo* without obvious side effects.

## Conclusion

In the present study, we developed muscone and RI7217 co-modified DTX long-circulating liposomes for anti-glioma treatment. RI7217 monoclonal antibody with high affinity and high selectivity for TfR in the brain achieve brain targeting through Tf‑mediated BBB penetration to reach the tumor site. Muscone modification increased the BBB permeability. Muscone and RI7217 co-modified DTX long-circulating liposomes enhanced the anti-glioma efficacy* in vitro* and *in vivo*. Thus, it is apparent that muscone and RI7217 antibodies co-modification have synergistic effects. In summary, based on the combined effects of TCM aromatic resuscitation and modern receptor-targeted technology, muscone and RI7217 co-modified dual-targeting delivery is a promising strategy to glioma chemotherapy.

## Supplementary Material

Supplementary figure.
