# Supplementary material for: Muscone/RI7217 co-modified upward messenger DTX liposomes enhanced permeability of blood-brain barrier and targeting glioma
Source: Theranostics. 2020 Mar 4;10(10):4308–22. doi: 10.7150/thno.41322 (PMC7150489; doi:10.7150/thno.41322)
Supplement: Supplementary file 1 — Supplementary figure. [file thnov10p4308s1.pdf]

Supporting Information

Muscone/RI7217 co-modified upward messenger DTX liposomes enhanced permeability of blood–brain barrier and targeting glioma

Shuangming Kang[1]*，Wenjuan Duan[2]*，Shangqian Zhang [2]， Dawei Chen [1✉], Jianfang Feng [3✉], Na Qi [2✉]

1 Department of Pharmaceutics, Shenyang Pharmaceutical University, Shenyang, 110016，China;

2 Department of Pharmacy, Guilin Medical University, Guilin, 541004，China;

3 Department of Pharmacy ,Guangxi University of Chinese Medicine, Nanning, 530299，China;

*These authors contribute equally to this work.

✉Corresponding author.： Tel/Fax: +86-773-5891498，E-mail: qina1012@glmc.edu.cn (Na Qi). fengjianfang@vip.163.com(Jianfang Feng). chendawei@syphu.edu.cn (Dawei Chen).

Supporting Information Figure of Contents Page

Figure.S1 (A) Chemical diagram of Muscone and DSPE-PEG$_{2000}$-NHS. (B) Flight Mass Spectrum of DSPE-PEG$_{2000}$-Muscone. (C) ^{1}H NMR spectrum of DSPE-PEG$_{2000}$-Muscone **S-2**

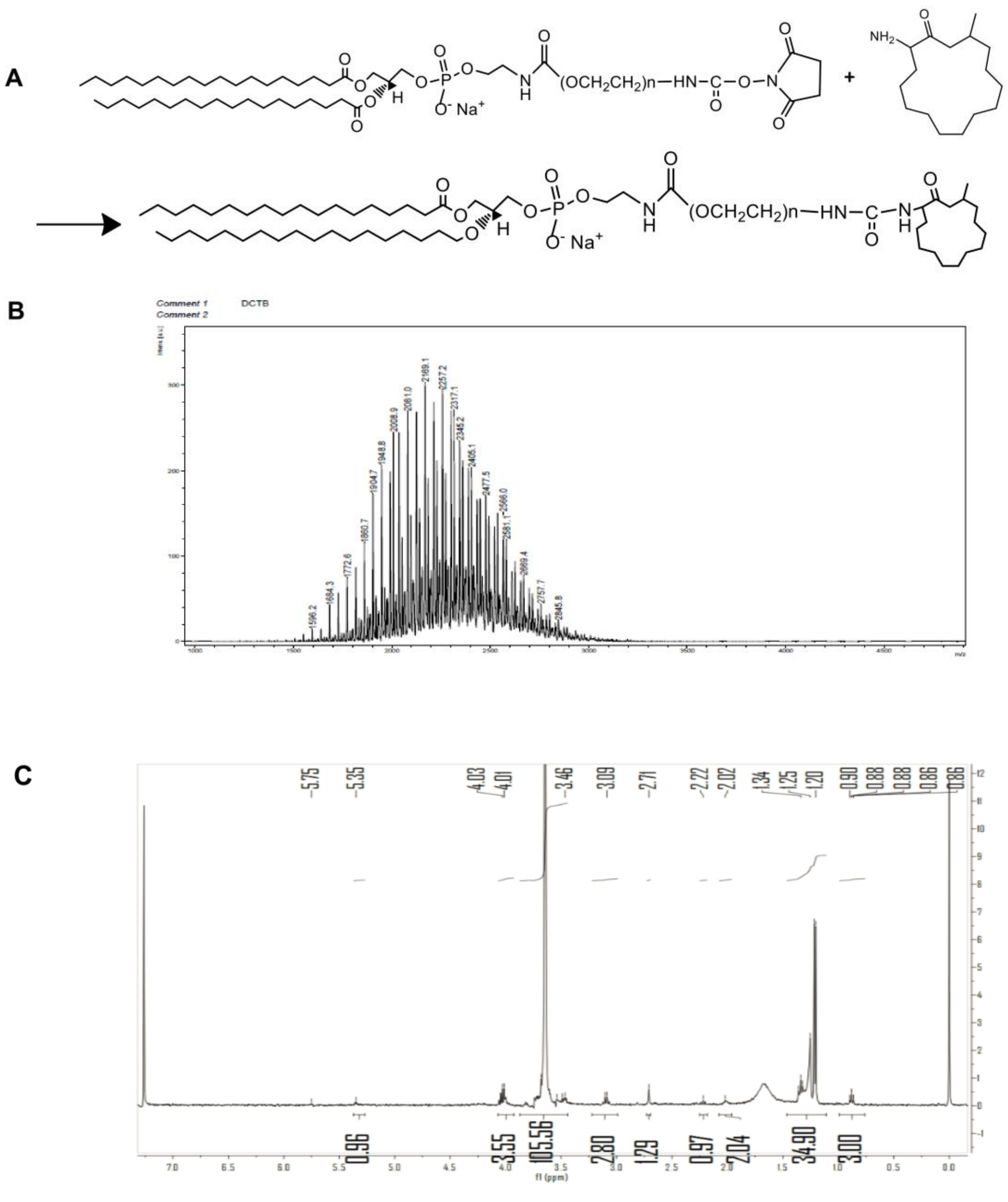

Figure.S1 (A) Chemical diagram of Muscone and DSPE-PEG$_{2000}$-NHS. (B) Flight Mass Spectrum of DSPE-PEG$_{2000}$-Muscone. (C) ^{1}H NMR spectrum of DSPE-PEG$_{2000}$-Muscone
